# National insights into patient safety culture in Chinese psychiatric hospitals: the role of hospital-level disparities

**DOI:** 10.3389/frhs.2026.1738071

**Published:** 2026-02-02

**Authors:** Yanhua Qu, Jing Shao, Xiaohong Li

**Affiliations:** Beijing Huilongguan Hospital, Peking University Clinical Medical School, Beijing, China

**Keywords:** hospital level, HSOPSC, mental health nurses, patient safety culture, psychiatric settings

## Abstract

**Background:**

Patient safety culture (PSC) is a critical component of healthcare quality, particularly in psychiatric settings where unique risks exist. In China, research on PSC within mental health institutions remains underdeveloped, and the factors influencing it are poorly understood.

**Methods:**

This cross-sectional study utilized the Chinese version of the Hospital Survey on Patient Safety Culture (HSOPSC) to assess PSC among 2,524 mental health nurses from multiple Level-2 and Level-3 psychiatric hospitals in China (2021–2023). We specifically examined the impact of hospital level on PSC perceptions. Multivariate regression models were employed to identify predictors of PSC composites.

**Results:**

The overall positive response rate (PPR) across the 12 PSC dimensions was 62.4%, indicating a moderate culture. Strengths were “Teamwork Within Units” (PPR = 83.3%) and “Organizational Learning” (PPR = 82.8%), while critical areas for improvement were “Nonpunitive Response to Error” (PPR = 46.6%) and “Staffing” (PPR = 43.3%). Regression analyses revealed that hospital level was a significant predictor of PSC outcomes, alongside years of experience and daily overtime hours (*P* < 0.05).

**Conclusions:**

This pioneering national study reveals that the PSC landscape in Chinese psychiatric hospitals is characterized by specific strengths but also critical weaknesses, significantly influenced by hospital level. The findings compel a move away from one-size-fits-all approaches. We recommend stratified interventions: foundational support for Level-2 hospitals and advanced quality initiatives for Level-3 hospitals, with universal prioritization of addressing staffing shortages and fostering a nonpunitive “Just Culture”.

## 1 Introduction

Patient safety is a critical component of healthcare quality, focused on minimizing preventable medical harm to an acceptable minimum (1, 2). The Institute of Medicine (IOM) has emphasized that safety is the most fundamental requirement for high-quality medical care (3, 4). In response, healthcare systems worldwide have implemented various strategies to enhance patient safety, including the development of safety guidelines, comprehensive training programs, and the fostering of a robust safety culture within hospitals (5–7).

Patient safety in psychiatric settings, however, presents unique challenges distinct from general healthcare. These challenges include managing risks of aggression and violence, the use of coercive measures, high levels of stigma, and the critical importance of fostering a therapeutic rather than a purely controlling environment (8–11). Given these complexities, cultivating a strong Patient Safety Culture (PSC), defined as shared values and behaviors focused on safety, becomes paramount for mitigating risks and ensuring a safe, therapeutic milieu (12). Evidence suggests that a psychologically safe environment can significantly enhance therapeutic relationships and reduce incidents of aggression (10, 11). Despite this, a significant practice gap remains, as many psychiatric nurses underutilize collaboration with patients to foster such an environment (13). International bodies like the WHO and IOM consistently highlight a robust PSC—encompassing leadership, teamwork, communication, and a nonpunitive approach to error—as a fundamental strategy for enhancing safety (14).

The Hospital Survey on Patient Safety Culture (HSOPSC) is a widely validated tool for measuring PSC (15, 16). Although numerous studies have assessed PSC across various clinical settings (17–20), research specifically focusing on nurses' perceptions within psychiatric care, particularly in China, remains limited (21–23). China's period of rapid development has been accompanied by rising psychological pressures, posing significant challenges to its healthcare system, especially in the realm of mental health patient safety (24). Furthermore, the Chinese healthcare system is characterized by a distinct hierarchical structure, wherein hospitals are formally classified into tiers (Level-1, Level-2, and Level-3) based on scale, technological sophistication, and service complexity. This classification is intrinsically linked to disparities in resource allocation, funding, and management capacity. Level-3 hospitals, as tertiary referral centers, typically benefit from greater resources and specialized expertise, whereas Level-2 hospitals, serving as regional centers, often operate with more constrained capacities. It is plausible that these systemic differences significantly influence the development and perception of PSC, yet this remains an unexamined area in psychiatric settings.

To address this critical evidence gap, this national, exploratory study aims to systematically describe and compare mental health nurses' perceptions of PSC across different hospital levels in China. By providing a comprehensive overview, this research seeks to identify key areas for improvement and deliver actionable insights for targeted interventions.

## 2 Methods

### 2.1 Study design and participants

A cross-sectional study was conducted. A convenience sample was recruited of 2,624 mental health nurses from psychiatric hospitals across seven regions in China. The participating hospitals were located in seven major geographic regions of China (e.g., North China, South China, East China, etc.), ensuring a broad geographic coverage that enhances the national representativeness of the sample. In the Chinese healthcare system, hospitals are officially classified into levels (Level-1, Level-2, and Level-3) based on their scale, technological sophistication, and service complexity, which is associated with differential resource allocation and management capacity. The present study deliberately excluded Level-1 hospitals (which are primary care institutions that typically lack specialized psychiatric inpatient units) and focused solely on Level-2 and Level-3 hospitals. This strategy was adopted to ensure homogeneity in the care setting and to facilitate a valid comparison of PSC perceptions between the two higher-level hospitals that form the backbone of China's specialized psychiatric inpatient care. Level-3 hospitals represent tertiary referral centers with greater resources and specialized expertise, while Level-2 hospitals serve as regional centers with more general services. This distinction provides important context for interpreting the observed disparities in PSC perceptions. Inclusion criteria comprised: (1) registered nurses working in psychiatric inpatient settings, (2) at least six months of tenure in their current unit. Participants completed the survey anonymously via the Wenjuanxing platform. After excluding 100 participants due to incomplete data or not meeting the inclusion criteria, the final analytical sample comprised 2,524 nurses.

### 2.2 Measures

The survey instrument comprised two main sections: (1) demographic and work-related variables, and (2) the Chinese version of the Hospital Survey on Patient Safety Culture (HSOPSC). The HSOPSC is a widely validated tool containing 42 items that measure 12 composites, encompassing 10 safety culture dimensions and 2 outcome dimensions (15). Items are scored on a five-point Likert scale. Following established methodology, the percentage of positive responses (PPR) for each dimension was calculated (15, 25).

We used a validated Chinese adaptation of the HSOPSC, which has been extensively applied in prior research within the Chinese context (24, 26, 27). This questionnaire was distributed as an anonymous online survey to the defined study sample via the Wenjuanxing platform between October 2019 and October 2023.

### 2.3 Statistical analysis

Data analysis was performed using SPSS software (version 25.0). Descriptive statistics were used to summarize participant characteristics and the Percent Positive Response (PPR) for each safety culture dimension. Continuous variables with normal distribution are presented as mean ± standard deviation (SD), while those with skewed distribution are presented as median (interquartile range), and categorical variables as frequencies and percentages (%). The PPR was calculated as the percentage of positive responses (e.g., “agree/strongly agree” or “always/most of the time”) for each dimension following established methodology (15).

The choice of specific statistical tests was guided by the nature and distribution of the data. Multiple linear regression analysis was employed to identify predictors of the outcome dimensions (“Overall Perception of Safety” and “Frequency of Events Reported”) while controlling for potential confounders. For subgroup analyses, non-parametric tests were selected as the PPR data were not normally distributed. The Kruskal–Wallis *H*-test was used to compare PPR across the two hospital levels (Level-2 vs. Level-3) for each of the 12 dimensions, and the Mann–Whitney *U*-test was used for comparisons between genders.

For the regression models, model fit was assessed using the Akaike Information Criterion (AIC) and Bayesian Information Criterion (BIC), with lower values indicating a better fit (28). Multicollinearity was checked via the variance inflation factor (VIF), and a value of less than 5 was considered acceptable (29). Statistical significance was set at a *p*-value of less than 0.05 for all tests.

### 2.4 Ethical consideration

Ethical approval was obtained from the Ethics Committee of the University (Project Identification Code: 20180206). Participation was anonymous and voluntary.

## 3 Results

### 3.1 Sample characteristics

A total of 2,524 participants from Level-2 and Level-3 hospitals were included in final analysis. The sample was predominantly female (87.2%), with a mean age of 32.1 years (SD = 7.6). Over half of the participants held an undergraduate degree (58.9%), and the majority were married (69.5%). Regarding professional experience, 22.1% had 1–5 years of experience, and 24.0% had 6–10 years. A substantial proportion of nurses (55.3%) reported working more than 30 min of overtime per day. The detailed demographic and work-related characteristics of the participants are summarized in Table 1.

**Table 1:** Demographics and work-related characteristics of participants (*N* = 2,524).

Characteristic	*n* (%)
Gender	
Female	2,201 (87.2%)
Male	323 (12.8%)
Education	
College or below	1,036 (41.1%)
Undergraduate	1,488 (58.9%)
Marital status	
Married	1,754 (69.5%)
Unmarried	770 (30.5%)
Years of experience	
1–5 years	558 (22.1%)
6–10 years	606 (24.0%)
>10 years	1,360 (53.9%)
Daily overtime	
≤30 min	1,128 (44.7%)
>30 min	1,396 (55.3%)
Hospital level	
Level-2	1,226 (48.6%)
Level-3	1,298 (51.4%)

### 3.2 Mental health nurses' perception about PSC

The analysis of PPR across the 12 PSC dimensions revealed substantial variation (Table 2). Based on established criteria (25), three dimensions were identified as strengths (PPR≥75%): “Teamwork within units” (83.3%), “Organizational learning/continuous improvement” (82.8%), and “Feedback and communications about error” (75.8%). Conversely, three dimensions, with PPR below 50%, were categorized as requiring improvement: “Staffing” (43.3%), “Nonpunitive response to error” (46.6%), and “Frequency of events reported” (49.3%). The remaining six dimensions had PPR ranging from 50% to 75%.

**Table 2:** The percent positive ratings (PPR) of safety culture dimensions.

Dimension	PPR (%)	Performance category
Teamwork within units	83.30	Strength
Organizational learning/continuous improvement	82.80	Strength
Feedback and communications about error	75.80	Strength
Supervisor/manager expectations & actions	72.50	Moderate
Hospital management support for patient safety	66.70	Moderate
Hospital handoffs & transitions	65.90	Moderate
Communication openness	62.50	Moderate
Overall perceptions of safety	59.20	Moderate
Teamwork across hospital units	56.40	Moderate
Frequency of events reported	49.30	Needs improvement
Nonpunitive response to error	46.60	Needs improvement
Staffing	43.30	Needs improvement

The performance categories were defined as follows: Strength (PPR ≥75%), Moderate (50% ≤PPR <75%), Needs Improvement (PPR <50%), according to established criteria (25).

### 3.3 Subgroup analysis by hospital level

The Kruskal–Wallis *H*-test revealed significant differences in PPR between Level-2 and Level-3 hospitals across several patient safety culture dimensions (Table 3). Level-3 hospitals demonstrated significantly higher PPR than Level-2 hospitals in five dimensions, including “Feedback and Communication about Error”, “Communication Openness”, and “Hospital Management Support for Patient Safety”. In contrast, for the dimension “Nonpunitive Response to Error”, Level-2 hospitals had a marginally higher PPR, but this difference was not statistically significant (*P* = 0.451).

**Table 3:** Comparison of PPR between hospital levels.

Dimension	Level-2 PPR (%)	Level-3 PPR (%)	*P*-value
Teamwork within units	82.1	84.5	0.125
Organizational learning/continuous improvement	81.2	84.3	0.058
Feedback and communications about error	72.1	79.3	**0** **.** **003**
Supervisor/manager expectations & actions	70.8	74.0	0.104
Hospital management support for patient safety	62.3	70.8	**<0** **.** **001**
Hospital handoffs & transitions	64.5	67.2	0.211
Communication openness	58.9	65.9	**<0** **.** **001**
Overall perceptions of safety	55.6	62.6	**<0** **.** **001**
Teamwork across hospital units	55.1	57.6	0.277
Frequency of events reported	45.8	52.6	**0** **.** **002**
Nonpunitive response to error	47.5	45.8	0.451
Staffing	42.1	44.4	0.327

Bold values indicate statistical significance (*P* < 0.05).

### 3.4 Predictors of patient safety culture

The multiple regression analysis (Table 4) showed that eight dimensions of PSC were significant predictors of “Overall Perceptions of Safety”, explaining 15.8% of the variance. For “Frequency of Events Reported”, gender, years of experience, and three safety culture dimensions were significant predictors, collectively accounting for 41.2% of the variance. Specifically, daily overtime hours were a significant negative predictor of the overall PSC score [*β* = −0.22, 95% CI (−0.38, −0.06), *P* = 0.007].

**Table 4:** Factors influencing the “overall perception of safety” and “frequency of events reported” in psychiatric settings.

Parameter	Overall perception of safety^a^ (*n* = 2,524)				The frequency of events reported^a^ (*n* = 2,524)				Collinearity	
	Estimate	*p*	95% CI		Estimate	*p*	95% CI			
			Lower	Upper			Lower	Upper	Tolerance	VIF^b^
Intercept	.666	<.001	.484	.848	1.854	<.001	1.477	2.231		
Gender	.005	.720	−.039	.057	−.040	.027*	−.211	−.012	.958	1.044
Education level	.031	.053	<.001	.056	−.024	.210	−.095	.021	.891	1.122
Marital status	−.024	.175	−.066	.012	−.032	.139	−.142	.020	.700	1.429
Staff position	−.034	.050	−.079	<.001	.022	.288	−.038	.127	.722	1.385
Years in hospital	.001	.952	−.013	.014	−.047	.045*	−.058	−.001	.578	1.731
Overtime per day	.013	.424	−.008	.019	−.005	.787	−.032	.024	.901	1.109
Teamwork within units	.030	.148	−.009	.062	−.044	.072	−.142	.006	.534	1.873
Staffing	.175	<.001*	.113	.178	.050	.035*	.005	.141	.555	1.803
Organizational learning/continuous improvement	.073	.001*	.033	.121	−.010	.696	−.110	.073	.501	1.996
Nonpunitive response to error	.079	<.001*	.031	.088	−.040	.087	−.111	.007	.597	1.676
Supervisor/manager expectations and actions promoting patient safety	.146	<.001*	.094	.173	−.018	.496	−.111	.054	.456	2.191
Feedback and communications about error	.060	.003*	.016	.079	.397	<.001*	.048	.616	.552	1.812
Communication openness	<.001	.985	−.034	.033	.027	.274	−.031	.109	.505	1.979
Hospital management support for patient safety	.144	<.001*	.083	.161	.049	.078	−.008	.153	.412	2.430
Teamwork across hospital units	.050	.048*	<.001	.090	.024	.426	−.055	.131	.356	2.812
Hospital handoffs and transitions	.135	<.001*	.075	.139	−.071	.004*	−.165	−.031	.520	1.922
Whole model summary	R^2^ = 0.161, Adjusted R^2^ = 0.158 F = 50.303, *P* < .001				R^2^ = 0.415, Adjusted R^2^ = 0.412 F = 185.031, *P* < .001					

All VIFs <5, showing no collinearity among the independent variables.

a Multiple linear regression model.

b VIF, Variance inflation factor.

* Represents the dimensions and items with statistical significance in psychiatric settings.

## 4 Discussion

This national study provides a comprehensive overview of PSC in Chinese psychiatric settings, revealing a culture with distinct strengths and vulnerabilities. It yields two fundamental contributions: (1) establishing crucial baseline measurements of PSC in a previously understudied context, and (2) novel identification of statistically significant disparities in PSC perceptions between different hospital tiers. These findings suggest that PSC development is not uniform but is potentially mediated by systemic and resource-dependent factors associated with hospital hierarchy. Consequently, enhancing PSC requires precisely targeted, stratified intervention strategies rather than generalized approaches.

### 4.1 Key drivers of PSC deficits: staffing and a culture of fear

Our results corroborate extant international literature, confirming that “Staffing”, “Nonpunitive Response to Error”, and the correlated “Frequency of Events Reported” represent pervasive and interconnected challenges in psychiatric settings (20, 22, 30).

#### 4.1.1 The staffing crisis: a fundamental prerequisite for safety

The critically low PPR for “Staffing” (43.3%), compounded by our finding that over half of the nurses worked substantial daily overtime, reveals a potentially dangerous operational norm. This state of chronic overextension directly threatens patient safety by impairing clinical vigilance and cognitive function, and is a well-established driver of professional burnout and psychological morbidity among healthcare workers (31, 32). The regression analysis, which identified “Staffing” as a significant predictor for both safety perceptions and reporting frequency, further solidifies this concern. This positions adequate staffing not as an operational luxury, but as a fundamental prerequisite for a functioning safety culture, mandating urgent prioritization in health policy and institutional resource allocation.

#### 4.1.2 From a culture of fear to a just culture

The persistently low scores for “Nonpunitive response to error” signify a profound cultural barrier that formal policies alone have failed to address. This likely stems from a deeply ingrained “culture of fear”, wherein practitioners anticipate reprisal despite nonpunitive mandates, a phenomenon noted previously (33). This environment suppresses the reporting of minor errors and near-misses, thereby crippling the organizational learning loop essential for proactive risk mitigation. Our findings underscore that moving beyond nominal policies to actively engineer a “Just Culture” is imperative (34). This requires clear institutional frameworks that distinguish between human error, at-risk behavior, and reckless conduct, thereby fostering trust, psychological safety, and continuous systemic learning.

### 4.2 The moderating role of hospital level: a call for stratified interventions

The subgroup analysis revealed statistically significant disparities in PSC perceptions between Level-2 and Level-3 hospitals. Level-3 hospitals demonstrated superior performance across key dimensions such as “Hospital management support”, “Communication openness”, and “Feedback and communication”, underscoring the influence of underlying structural determinants associated with hospital hierarchy.

This divergence may be attributed to a multifactorial etiology. First, disparities in financial and institutional resources likely enable Level-3 hospitals to implement more robust safety-specific training and infrastructure (26). Second, variations in managerial sophistication and leadership practices may foster greater transparency and supportive supervision in Level-3 settings. Third, differences in human capital, as evidenced by the higher proportion of degree-qualified and experienced nurses in Level-3 hospitals (Table 1), may contribute to a workforce more adept in systems-based safety practices.

These findings carry significant policy implications, indicating that PSC enhancement initiatives must be stratified. Interventions in Level-2 hospitals should concentrate on foundational elements, such as strengthening leadership engagement, establishing basic communication protocols, and implementing nonpunitive reporting systems. In contrast, Level-3 hospitals can leverage their advantages to focus on advanced, iterative quality improvement and sophisticated root-cause analysis.

Finally, while our regression models were statistically significant, the modest explained variance (Adjusted R^2^= 15.8% for “Overall Perceptions of Safety”) warrants comment. This is consistent with the complex, multifactorial nature of PSC, which is influenced by numerous factors beyond our model's scope (35). In contrast, the model for the “Frequency of Events Reported” accounted for a substantially higher proportion of variance (Adjusted R2 = 41.2%), suggesting that the predictors in our framework are more strongly associated with this behavioral outcome than with global perceptions. Despite this difference, the remaining unexplained variance for both outcomes underscores the influence of other unmeasured contextual or individual factors. Nonetheless, the primary value of our analysis lies in identifying significant and actionable predictors, such as staffing and nonpunitive response to error, which remain crucial for targeted interventions.

### 4.3 Limitations and future research

This study has several limitations that should be considered when interpreting the findings. First, its cross-sectional design precludes the inference of causality. Second, the deliberate exclusion of Level-1 hospitals, while necessary to ensure sample homogeneity, limits the generalizability of our findings to the entire spectrum of psychiatric hospitals in China. Third, the exclusive reliance on nurses' perspectives omits the viewpoints of other clinical and non-clinical staff (e.g., physicians, aides, administrators), which would provide a more holistic cultural assessment. Finally, although our sample of 2,524 nurses from multiple regions is substantial, the use of a convenience sampling method may limit the generalizability of the findings to all psychiatric nurses in China and could introduce selection bias. Therefore, while the large sample size is a strength, the findings should be generalized to the broader national context of Chinese psychiatric nurses with caution.

Based on these limitations, future research should prioritize several directions. First, and foremost, while the large sample size is a strength, the findings should be generalized with caution. Nationally representative studies employing probability sampling methods are warranted to enhance the external validity of the findings. Second, longitudinal or mixed-methods designs are needed to elucidate the causal mechanisms underlying the inter-hospital disparities identified here. Third, there is a particular need for intervention studies that develop and evaluate tiered improvement strategies tailored to the specific needs and resources of different hospital levels. Finally, future investigations should explore the role of specific leadership behaviors and organizational climates that mediate the relationship between hospital level and PSC.

## 5 Conclusions

This national study provides critical insights into the PSC within Chinese psychiatric hospitals, revealing a landscape characterized by both universal challenges and significant disparities linked to hospital hierarchy. The findings compellingly demonstrate that effective PSC enhancement necessitates differentiated, precisely targeted strategies rather than a one-size-fits-all approach.

Healthcare policymakers, nursing administrators, and institutional managers must therefore prioritize stratified actions. First, addressing the staffing crisis through advocacy for national policy reforms on nurse-patient ratios and overtime regulations represents a non-negotiable foundation for safety across all institutions. Second, actively cultivating a nonpunitive “Just Culture” is essential to break the cycle of fear and under-reporting, thereby transforming errors into opportunities for systemic learning. Finally, interventions must be tailored to the hospital level: Level-2 hospitals should focus on strengthening core management support and foundational communication processes, while Level-3 hospitals can leverage their advantages to pursue advanced safety practices and innovation.

Through these coordinated, system-level actions, healthcare institutions can systematically strengthen patient safety culture, ultimately fostering a safer and more reliable therapeutic environment for both patients and healthcare professionals.

## Data Availability

The datasets presented in this article are not readily available because Data are subject to privacy and ethical restrictions to protect patient confidentiality. They are available from the corresponding author upon reasonable request for verification of research findings, subject to a signed confidentiality agreement. Requests to access the datasets should be directed to yanhuaqu, quyanhuar@126.com.
